# Cost-effectiveness of Left Ventricular Assist Devices (LVADs) as destination therapy in the UK: An economic modelling study

**DOI:** 10.1371/journal.pone.0312912

**Published:** 2024-12-04

**Authors:** Tuba Saygın Avşar, Louise Jackson, Pelham Barton, Sophie Beese, Hoong S. Lim, David Quinn, Malcolm Price, David J. Moor

**Affiliations:** 1 Department of Applied Health Research, University College London, London, United Kingdom; 2 University of Birmingham, Birmingham, United Kingdom; 3 University of Birmingham Hospitals, Birmingham, United Kingdom; 4 Department of Public Health, Canadian University Dubai, Dubai, United Arab Emirates; University of Naples Federico II, ITALY

## Abstract

**Objectives:**

Left Ventricular Assist Devices (LVADs) for destination therapy (DT) are used in many countries but in some, like the UK, LVADs are not commissioned due to uncertainty around their cost-effectiveness. Existing economic evaluations of LVADs for these patients have limitations. This study aimed to estimate the cost-effectiveness of LVADs as destination therapy, compared to optimal medical therapy, in the UK.

**Methods:**

A cost-utility analysis from a UK healthcare perspective was conducted, using a Markov model. The model incorporated the impact of major events and complications. Sub-group analyses considered different severities of heart failure on cost-effectiveness. Uncertainty was measured in deterministic and probabilistic sensitivity analyses.

**Results:**

LVAD produced additional 2.78 (95% CI 2.46–3.14) QALYs at an incremental cost of £152,329 (95% CI £125,665 - £181,812) compared to medical management, giving an incremental cost-effectiveness ratio (ICER) of £54,748 per QALY. The ICER remained above the accepted thresholds of cost-effectiveness in the UK if a small proportion of patients receiving LVAD becomes eligible for a heart transplant and for all subgroups based on heart failure severity. The deterministic sensitivity analysis showed that the ongoing outpatient costs had a significant impact on the results.

**Conclusions:**

Our analysis found that LVADs are not cost-effective as destination therapy in the UK if a willingness to pay threshold of £50,000 per QALY gained or disease severity modifiers, were applied. Robust data on ongoing costs for LVAD and medical management are needed.

## 1. Introduction

Heart failure (HF) is defined as ‘a disease characterised by a decline in the heart’s ability to pump blood around a person’s body at normal filling pressures to meet its metabolic needs’ [1]. Advanced heart failure (AHF) is characterised by severe symptoms including shortness of breath at rest or with minimal exertion, cachexia and muscular deconditioning, refractory fluid overload and kidney and liver failure despite the use of conventional HF medications [2].

The economic burden of HF to healthcare systems is considerable; for example the direct cost of HF to the UK National Health Service (NHS) was estimated at £716 million annually, accounting for approximately 2% of the NHS budget [3]. Similarly, a recent review of the medical costs associated with HF in the US estimated that annual median total costs for HF care were $24,383 per patient in 2019 prices [4]. Heart transplantation is the main therapy for patients AHF. However, patients not suitable for heart transplantation might need left ventricular assist devices (LVAD) as destination therapy (DT). The objective of LVAD as DT is to provide symptomatic and prognostic benefit to patients with AHF who are at high risk of mortality on medical therapy and not suitable for heart transplantation. Whilst LVADs for DT are used in many countries, currently they are not funded in the UK NHS due to a lack of evidence on their cost-effectiveness; however, LVAD can be funded as bridge-to-transplant (BTT) for patients who are candidates for heart transplantation but may be deteriorating on medical therapy whilst on the waiting list [5].

There have been three generations of LVADs, and life expectancy and quality of life have substantially improved with each generation. Also, the risks of complications associated with LVADs have reduced, including device and lead issues and hemocompatibility-related adverse events (HRAEs), such as stroke [6]. Our recent systematic review of all economic evaluations of LVADs as DT found that the estimates of incremental cost per additional quality-adjusted-life-year gained (ICER per QALY) varied widely between £238,401 and £47,361 in 2019 prices, with more recent studies tending to have lower ICERs due to the technology advances [7]. Important limitations in the evaluations were the omission of serious adverse events and the use of data from non-DT populations. Two recent economic evaluations gave similar ICER per QALY estimates which were above conventional thresholds of cost-effectiveness applied in the UK but in the region of thresholds that until recently could be applied under specific end-of-life criteria by NICE [8]. One of the evaluations is no longer relevant as it focussed on the withdrawn HVAD [9] and the other, whilst focussing on HM3 had limitations in terms of modelling assumptions and the short time horizon [10]. Given the uncertainty around the cost-effectiveness of LVAD for DT, which has limited informed decision-making around use in the UK NHS, a new economic evaluation was commissioned by the UK National Institute for Health and Care Research (NIHR128996).

## 2. Methods

A Markov model was designed to evaluate the cost-effectiveness of LVAD as destination therapy (DT) for patients who are ineligible for a heart transplant, compared to medical management (MM) from the UK NHS perspective. A Markov model was appropriate due to the substantial life expectancy impacts of advanced heart failure [4]. The model development was informed by clinicians, LVAD recipients and their family members, and LVAD related research.

### 2.1 Model description

**Fig 1:**
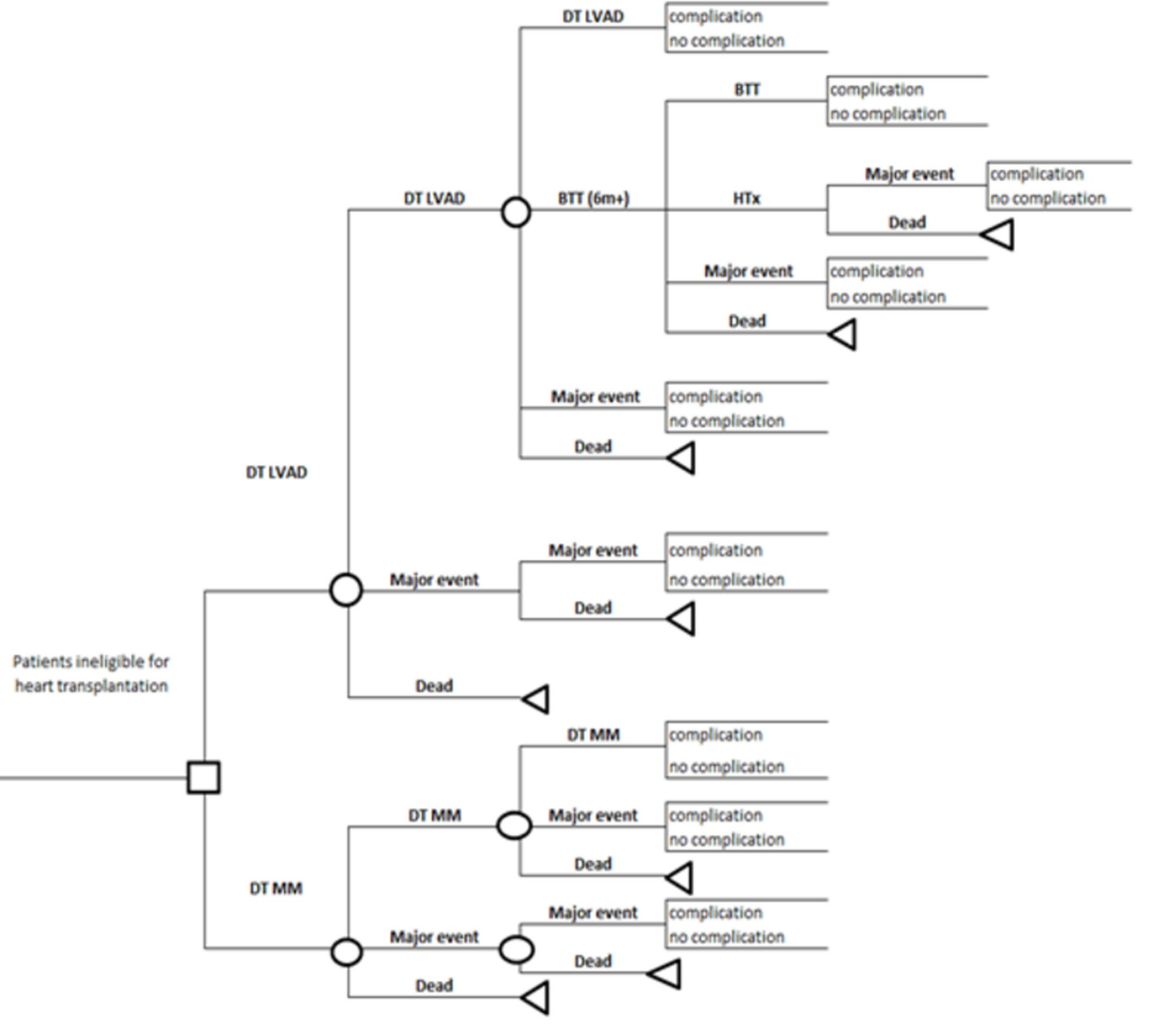
Structure of the LVAD model.

In the model (Fig 1), heart transplant ineligible patients receive LVAD while the comparator group receives MM. The model runs in monthly cycles, and at the end of each cycle, patients can be alive without any major event, alive with major events, or have died. Major events were those at substantially increased risk of mortality–stroke (non-disabling and disabling), aortic regurgitation, and right heart failure (RHF)–and were modelled as separate health states to incorporate their impacts on mortality and quality of life. The model structure incorporates the fact that LVAD recipients might experience any of these major events while MM patients might experience stroke. In the cases where patients experience more than one major event, the model uses the major event with the greatest impact on mortality and quality of life. For example, patients who previously experienced a stroke might experience a disabling stroke, and after that they would stay in the disabling stroke state until dying. In addition to the major events, patients who receive LVAD might also experience complications which include gastrointestinal bleeding (GIB), driveline infection (DI), pump exchange (PE), and arrhythmia. The probability of hospitalisations due to other reasons among MM patients is incorporated when estimating the cost implications. The transitions within the model are summarised in Table 1.

**Table 1:** Transitions within the model.

Starting state	Jump to state	Complications
**DT (LVAD)**	Death,	GIB, DF, DI, PI, PE, Arrhythmia
	Non-disabling stroke, disabling stroke, RHF, AR, DT (LVAD)	
**Non-disabling stroke**	Death	GIB, DF, DI, PI, PE, Arrhythmia
	Non-disabling stroke, disabling stroke, AR, RHF	
**Disabling stroke**	Death,	GIB, DF, DI, PI, PE, Arrhythmia
	disabling stroke.	
**RHF**	Death,	GIB, DF, DI, PI, PE, Arrhythmia
	Disabling stroke, RHF	
**AR**	Death,	GIB, DF, DI, PI, PE, Arrhythmia
	RHF, disabling stroke, AR	
**MM**	Death,	Re-admission for any reason
	Non-disabling stroke, disabling stroke	

DT LVAD: Destination therapy left ventricular assist device, RHF: Right heart failure, AR: Aortic regurgitation, GIB: Gastrointestinal bleeding, DF: Device failure, DI: Driveline infection, PI: Pump infection, PE: Pump exchange.

#### 2.1.1. Model inputs

Model inputs were identified from the published literature, and choices between different sources were made based on clinical expert views. The parameters used in the model are provided in Supplementary Material.

*2*.*1*.*1*.*1*. *Probabilities*. Mortality rates published by the Office for National Statistics (ONS) were used to obtain the age-standardised mortality risks [11]. The overall mortality risks for DT and OMM patients were adjusted for the probability of death due to a major event [12].The mortality risks reported for LVAD recipients in the MOMENTUM trial, which was a prospective, multicentre, nonrandomized study evaluating the HeartMate 3, and for MM patients in the REMATCH trial, the only trial that compared MM to LVAD, were utilised to obtain monthly probabilities [13, 14]. It was assumed that the profiles of patients in these two studies were relatively similar despite the time difference between when they were undertaken.

The probabilities for experiencing major events and complications were identified from the published literature (Supplementary Material).

*2*.*1*.*1*.*2*. *Quality of life*. The baseline utility value reported in the MOMENTUM trial was utilised for the MM patients and the utility values after an LVAD implant were used in the LVAD arm [14]. Utility decrements were applied to those who experienced a major event or complication. It was assumed that major events would cause a permanent utility loss, while complications would only reduce quality of life during the cycle in which the complication occurred.

*2*.*1*.*1*.*3*. *Costs and resource use*. The costs and resource use associated with the intervention and comparator were estimated using various sources. To identify the one-off expenses, for example, the costs associated with stroke, NHS reference costs were used for the operation costs while the number of hospital days in ICU and on a cardiac ward were estimated through discussions with two practising heart surgeons, working in the NHS. Ongoing cost inputs, such as the outpatient costs for LVAD recipients, were identified from a systematic review [7]. In the absence of up-to-date UK-based data on the ongoing costs of medical management, estimates in previous economic evaluations identified in that review were discussed with the clinical experts in the research team and judged to be reasonable albeit dated. All the costs were reported in 2019 GBP as this was the nearest full year date to the start of the modelling.

### 2.2 Population

The study population consisted of a hypothetical cohort of 1,000 patients with advanced heart failure who were ineligible for heart transplant. The mean age was 65 years because the mean age was reported as 63 in the key trial (MOMENTUM 3) and similar in other clinical trials. A review of the studies reporting clinical effectiveness of LVADs identified the female ratio as varying from 20% to 79% and did not identify any statistically significant impact of sex [7]. Therefore, 50% of the cohort was assumed to be female to reflect this evidence.

### 2.3 Outcomes and analysis

The primary outcomes were incremental costs per life year (LY) and QALY gained. For this, the healthcare costs, LYs and QALYs per patient were calculated for the OMM and LVAD arms. All the analyses were conducted over a lifetime horizon and over shorter time periods of two and five years. The costs and benefits were discounted at 3.5% as per National Institute for Health and Care Excellence (NICE) guidelines [8]. Cost-effectiveness was defined according to the NICE guidelines, which suggest health technologies with an ICER per QALY of between £20,000 and £30,000 are considered cost-effective in the UK [8]. Until recently NICE used an end-of-life criteria where the threshold could be raised to £50,000 per QALY gained when median survival for the comparator was less than 2 years and the intervention conveyed a benefit of at least 3 months additional survival. However, the recently updated guidelines recommend a severity-weighting based on a QALY shortfall estimate [8]. The absolute QALY shortfall is defined as the difference between the expected QALYs for a specific age and sex group in the general population and the expected QALYS for the patient population if treated with standard supportive NHS care. The proportional QALY is estimated by dividing the absolute QALY shortfall by the remaining QALYs for the same age and sex group in the general population. The QALY Shortfall Calculator developed by Sheffield University was used to estimate the QALY shortfalls in this study [15].

### 2.4 Deterministic and probabilistic sensitivity analysis

Deterministic and probabilistic sensitivity analyses were conducted to estimate uncertainties and the impact of each parameter. In the deterministic sensitivity analysis, key parameters were varied and the impacts on the model outcomes were presented graphically (Supplementary Material). The input values for the one-way sensitivity analysis were obtained from the studies identified in a systematic review and from two previous UK studies (9, 10). Additionally, the impact of incorporating the probability of transitioning to the BTT (0.006/m) and HT states (0.028) for LVAD patients was also estimated. Finally, a probabilistic sensitivity analysis (PSA) was conducted to estimate the uncertainties around the model outcomes. The parameters used in the sensitivity analyses are provided in Supplementary Material.

### 2.5 Exploratory subgroup analysis by INTERMACS profiles

The cost-effectiveness of LVAD based on the clinical characteristics of patients was analysed in an exploratory sub-group analysis, using the impact of severity of heart failure classification (INTERMACS), which classifies patients with advanced heart failure into one of seven categories based on clinical stability, functional capacity, and severity of symptoms. INTERMACS profiles 1 (Critical Cardiogenic Shock), 2&3 (Progressive Decline), and 4&5 (Exertion Intolerant) were used in this analysis. INTERMACS 1 was evaluated separately but not included in the base-case because DT patients in this group usually do not receive LVADs. The mortality risks and health utilities used for this analysis were defined based on the best available evidence and expert views (Supplementary Material). A lower INTERMACS profile was associated with greater frailty and lower quality of life.

## 3. Results

The model outcomes over different time horizons are provided in Table 2. Over a lifetime, LVAD produced an additional 2.86 QALYs at an incremental cost of £152,735, with the ICER per QALY gained was estimated as £53,496. It was £67,997 in over five years and over two years £131,593. The absolute QALY shortfall for the study population was estimated as 10.38, while the proportional QALY shortfall was 0.96. The appropriate weighting was 1.7, and the weighted ICER per QALY was calculated as £31,468 (Table 3). Therefore, although the QALY-weighting reduced the ICER per QALY estimate, it was still above the upper bound of NICE’s recommended cost-effectiveness threshold (£30,000 per QALY) (7). As the base-case ICER was above the upper limit of cost-effectiveness under the old NICE end-of-life criteria, the interpretation of the ICER being above the acceptable thresholds of NICE is consistent across assessment modalities.

**Table 2:** Deterministic outcomes.

Lifetime outcomes	MM	LVAD	Incremental
**Expected LYs per patient**	0.92	4.65	3.73
**Expected QALYs per patient**	0.46	3.32	2.86
**Cost per patient**	£18,886	£171,621	£152,735
**Incremental cost per LY**			£40,911
**Incremental cost per QALY**			**£53,496**
**2-year outcomes**	**MM**	**LVAD**	**Incremental**
**% of deaths**	91%	25%	-66%
**Expected LYs per patient**	0.85	1.71	0.87
**Expected QALYs per patient**	0.43	1.23	0.80
**Cost per patient**	£17,173	£121,843	£104,670
**Incremental cost per LY**			£120,868
**Incremental cost per QALY**			**£131,593**
**5-year outcomes**	**MM**	**LVAD**	**Incremental**
**% of deaths**	100%	54%	-46%
**Expected LYs per patient**	0.92	3.30	2.38
**Expected QALYs per patient**	0.46	2.36	1.90
**Cost per patient**	£18,855	£148,002	£129,148
**Incremental cost per LY**			£54,314
**Incremental cost per QALY**			**£67,997**

LY: Life years, QALY: Quality-adjusted life-years, MM: Medical management, LVAD: Left ventricular assist device

**Table 3:** Weighted ICER per QALY estimates.

Mean QALYs for MM	Proportional QALY shortfall	Recommended QALY weight	ICER per QALY	Weighted ICER per QALY
**0.46**	0.96	1.7	£53,496	£31,468

### 3.1 Deterministic sensitivity analysis

**Fig 2:**
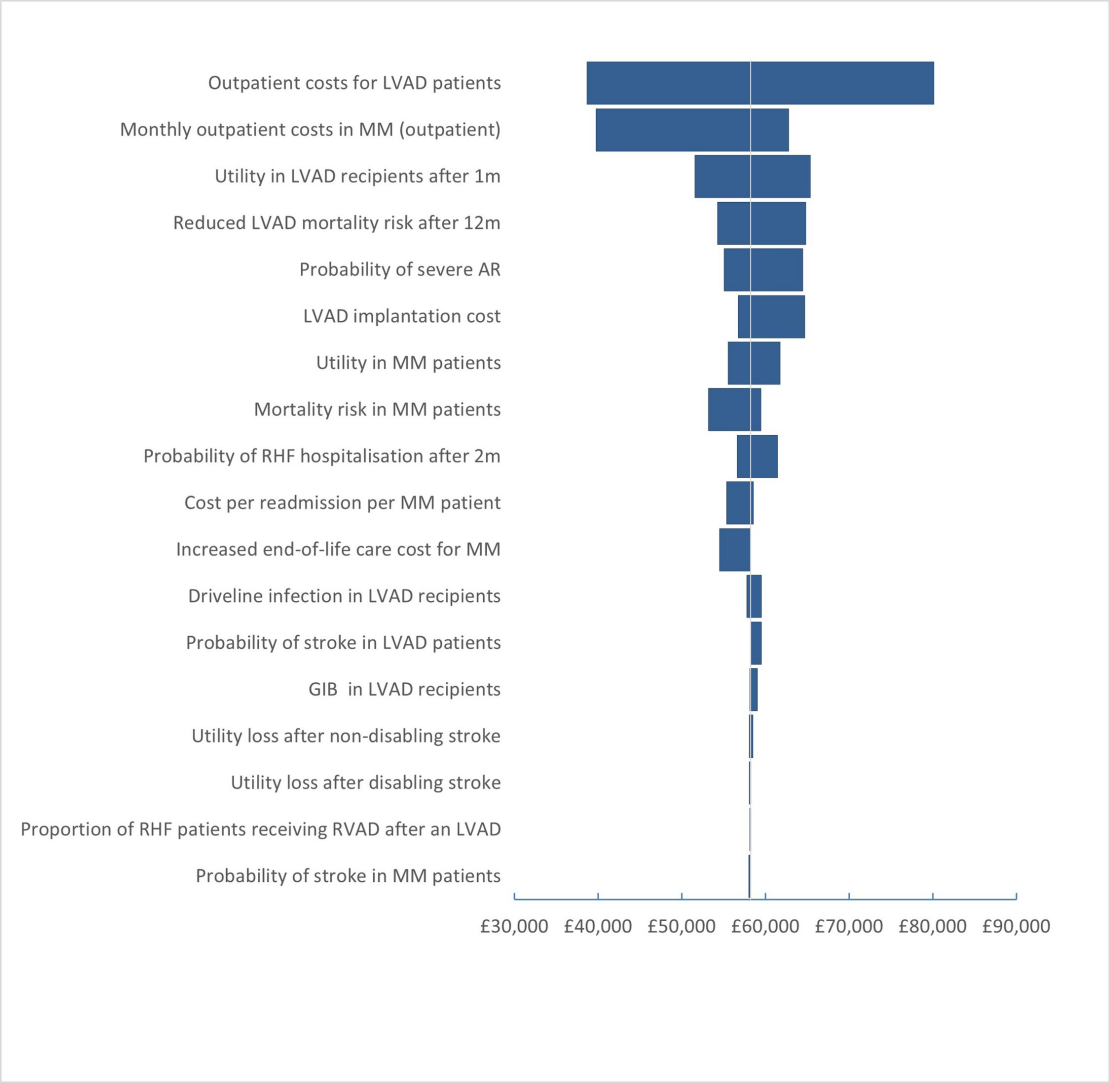
Tornado diagram of one-way sensitivity analysis.

The base-case analysis findings did not change in the deterministic sensitivity analysis. When the transition from DT to BTT states and from BTT to HT states was incorporated, the ICER reduced slightly from £53,496 to £52,762 per QALY 9 (Table 4). One-way sensitivity analysis defined that the outpatient costs for LVAD recipients had the greatest impact on the ICER estimates; ICER per QALY varied between £38,000 and £80,000 when different figures reported in the literature were applied. Similarly, the outpatient costs for patients on OMM and the mortality risk amongst LVAD patients had substantially impacted the ICER per QALY estimates (Fig 2). However, ICER per QALY remained higher than the accepted WTP thresholds of cost-effectiveness.

**Table 4:** Model outcomes when the transition to BTT and HT was incorporated.

Lifetime outcomes	MM	LVAD	Incremental
Expected LYs per patient	0.92	4.58	3.66
Expected QALYs per patient	0.46	3.26	2.80
Cost per patient	£18,886	£166,433	£147,547
Incremental cost per LY			£40,351
Incremental cost per QALY			**£52,762**

### 3.2 Probabilistic sensitivity analysis

**Fig 3:**
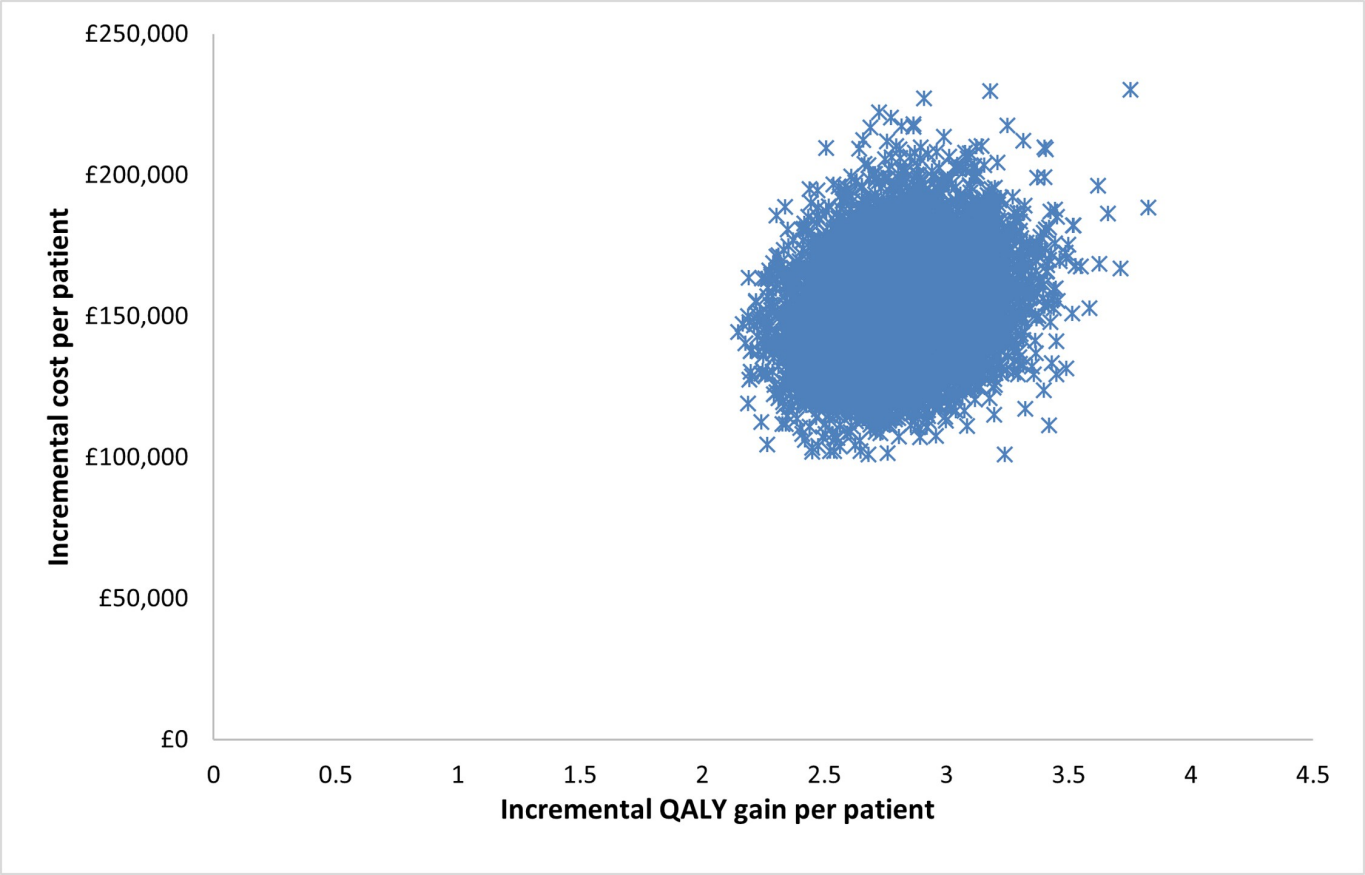
Probabilistic sensitivity analysis based on 10,000 iterations.

**Fig 4:**
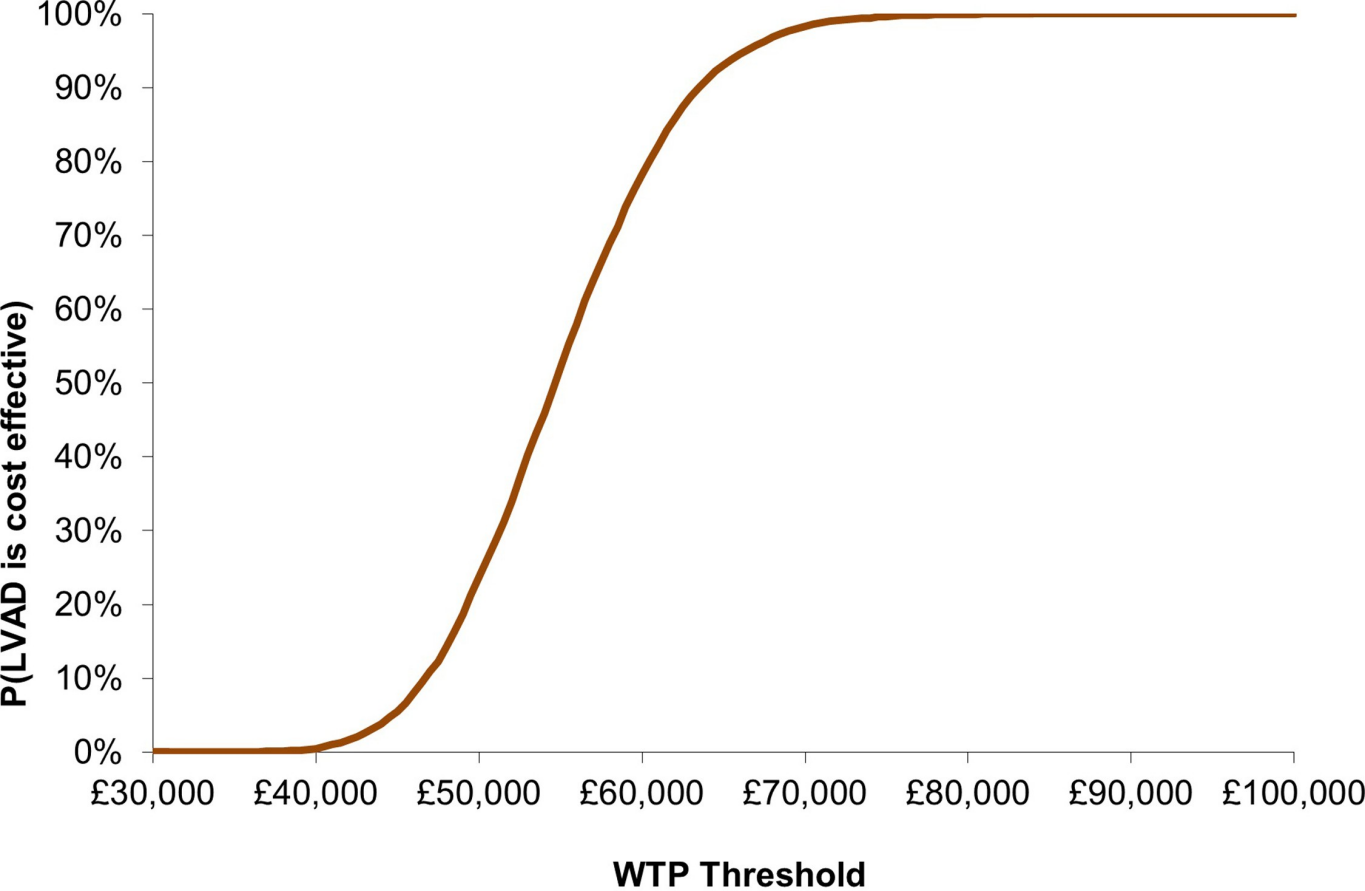
Cost-effectiveness acceptability curve (CEAC).

The probabilistic sensitivity analysis based on 10,000 iterations showed some uncertainty around the model outputs. The mean estimates and the 95% confidence intervals (CI) are provided in Table 5 and the ICER estimates are shown in Fig 3. The analysis also found that the probability of cost-effectiveness at a Willingness-to-pay (WTP) threshold of £30,000 was 0%, from around 10% at £40,000 to 90% at £60,000, reaching 100% at a WTP of £75,000. This indicates that the transition is stepped but away from any threshold of cost-effectiveness used in the UK even if adjusted by a severity weighting (Fig 4).

**Table 5:** Probabilistic sensitivity analysis outcomes.

	MM			LVAD			Incremental		
**Outcomes**	**Mean**	**95% CI**		**Mean**	**95% CI**		**Mean**	**95% CI**	
**Expected LYs per patient**	0.93	0.80	1.08	4.59	4.14	5.10	3.67	3.19	4.19
**Expected QALYs per patient**	0.48	0.41	0.55	3.26	2.94	3.61	2.78	2.46	3.14
**Cost per patient**	£18,953	£17,107	£21,016	£171,281	£144,725	£200,692	£152,329	£125,665	£181,812
**Incremental cost per QALY £54,748**									
**Probability of cost-effectiveness at £30,000 0%**									

### 3.3. Exploratory subgroup analysis by INTERMACS profiles

The sub-group analysis by INTERMACS profiles showed that patients in the LVAD arm who had less severe heart failure would gain more LYs and QALYs than patients with more severe conditions (Table 6). ICER per QALY estimates were £84,800, £65,458, and £58,815 for the INTERMACS groups 1, 2&3 and 4&5, respectively. Thus, the ICER remained above the £30,000 threshold in all cases. Important to note that these estimates are not directly comparable to the base-case outcomes, given the sources of the data needed for the INTERMACS profiles.

**Table 6:** Model outcomes by INTERMACS profiles.

Lifetime outcomes (INTERMACS 1)	MM	LVAD	Incremental
Expected LYs per patient	0.37	3.05	2.68
Expected QALYs per patient	0.06	1.65	1.59
Cost per patient	£13,333	£148,214	£134,882
Incremental cost per LY			£50,336
Incremental cost per QALY			**£84,800**
**Lifetime outcomes (INTERMACS 2&3)**	**OMM**	**LVAD**	**Incremental**
Expected LYs per patient	0.92	3.93	3.01
Expected QALYs per patient	0.36	2.54	2.17
Cost per patient	£18,886	£161,052	£142,166
Incremental cost per LY			£47,198
Incremental cost per QALY			**£65,458**
**Lifetime outcomes (INTERMACS 4&5)**	**OMM**	**LVAD**	**Incremental**
Expected LYs per patient	1.00	4.14	3.14
Expected QALYs per patient	0.51	2.96	2.45
Cost per patient	£19,714	£163,958	£144,244
Incremental cost per LY			£45,967
Incremental cost per QALY			**£58,815**

## 4. Discussion

This economic modelling study evaluated the cost-effectiveness of LVAD in the UK for advanced heart failure patients who are ineligible for a heart transplant. The analyses showed that LVAD would increase life expectancy by 3.67 (95% CI 3.19–4.19), produce an additional 2.78 QALYs (95% CI 2.46–3.14) and the incremental costs to the NHS would be £152,329 (95% CI £125,665 - £181,812) per person, resulting with an ICER of £54,748 per QALY. Thus, at a WTP of £30,000 per QALY, LVADs were not cost-effective compared to MM in DT patients in the UK. The sensitivity analyses showed that the inclusion of the probability of becoming eligible for a heart transplant did not change the findings, while the outpatient costs for LVAD recipients significantly impacted the ICER estimates. Some uncertainty was observed around the model outcomes in the probabilistic sensitivity analysis, especially in the incremental QALY gains. The probability of cost-effectiveness at a WTP of £30,000 was 0% based on 10,000 iterations. According to the exploratory sub-group analysis, LVAD was not cost-effective for any specific INTERMACS group evaluated.

A previous economic evaluation in the UK estimated an ICER of £47,361 per QALY gained [10]. The critical difference between that evaluation and the current study is related to the mortality risks assumed for LVAD recipients beyond 24 months. Lim et al. [10] extrapolated the values, averaging the changes in the last six months while, in the current study, the risk for the 24th month was used for the remaining cycles. When the extrapolated values used in that study were entered into the current model, the ICER per QALY estimates reduced from £54,295 to £49,120, as shown in the deterministic sensitivity analysis. In terms of the incremental costs, for the study by Lim et al. [10], the cost difference between the LVAD and medical management arm was only £113,552, which was mainly related to the cost of the device (£109,140). The corresponding figure in the current analysis was £152,735 and £146,275. Thus, the study by Lim et al. [10] assumed very little difference between patients on medical management and LVAD regarding adverse events and complications. The other recent study from the UK [9] reported a similar ICER per QALY gained but that study is not directly comparable due to focusing on an LVAD that is now withdrawn (HVAD).

This study has several strengths. Firstly, the model was developed utilising evidence from a robust systematic review of the literature and with input from clinical experts to ensure consistency with current practice. The analysis addresses some of the limitations associated with previous models, comprehensively incorporating evidence on adverse events, using data relating to relevant populations, alongside adopting an appropriate time horizon. The limitations arising from data scarcity and modelling assumptions should be considered. In the absence of trials that compared LVAD recipients to patients on medical management in terms of INTERMACS profiles, the best available evidence and expert views were used to define the key model inputs in this analysis. The model development was also informed by discussions with clinicians, commissioners, and patient representatives.

Since currently only one type of LVAD is available in the UK (HM3), the key model parameters were defined from the studies that included only this type of LVAD. However, device-specific data was unavailable for some major events and complications and data that included previous versions of LVAD were used for these parameter estimates. Thus, the model might be underestimating or overestimating the benefits of LVAD, depending on the parameters chosen. However, the deterministic sensitivity analysis showed that none of these parameters substantially impacted the model results.

The model used life expectancy data on MM published in 2001; thus, the estimates assumed that the clinical effectiveness of standard care has stayed the same over the last 20 years. Therefore, these results estimates might be overestimating the benefits of LVADs, if the life expectancy of OMM patients has improved over the previous 20 years. Additionally, it was assumed that the profiles of patients in the MOMENTUM trial matched the profiles of OMM patients in the REMATCH trial. This was a reasonable assumption given that the OMM patients in the REMATCH trial were on inotropes, and most LVAD recipients in the MOMENTUM trial were classified as INTERMACS 2&3. However, if this assumption is incorrect and OMM patients in the REMATCH trial had worse health status compared to the baseline health statuses of the patients in the MOMENTUM trial, the model outcomes might be overestimating the benefits of LVADs.

The mean age of the modelled population was 65 years, and this was chosen due to the availability of relevant LVAD trial data, and a paucity of data to model a population of greater age with possibly greater comorbidities and frailty. Should data on the latter become available analysis sub-grouped by age would be possible. Additionally, although the clinical studies that were used to develop the model reported sex-adjusted outcomes, they were usually not available by sex. Therefore, it was not possible to estimate cost-effectiveness by sex. The implications on this on the study outcomes is not clear. The model distinguishes males and females and uses different mortality rates. Therefore, should data on clinical effectiveness and adverse events by sex become available, the model can be updated to incorporate these.

Another consideration is that no trial compared LVAD to medical management for defined INTERMACS profiles. Hence, the sub-group analyses were based on discrete data from different sources, and assumptions were made based on expert views for some parameters. It is difficult to speculate on the impacts of these assumptions on study findings in the absence of data; therefore, the sub-group analyses should be considered exploratory. The sub-group analyses were based on the best available evidence and expert views were consistent with the base-case. However, the existing evidence was insufficient to reach a full conclusion on the cost-effectiveness of LVADs for patients with different INTERMACS profiles. Further research is needed to estimate the impacts of INTERMACS profiles on the cost-effectiveness estimates.

The sensitivity analyses indicated that varying the outpatient costs for patients on LVAD and medical management significantly impacted the results. Thus, future research focusing on determining these cost items might be helpful for decision-makers in the UK. In particular, the cost of OMM requires further investigation to consider the increased use of inotropes, which require frequent hospital admissions, mitral and tricuspid valve clip implantation, implantable cardioverter-defibrillators, cardiac resynchronization therapies, and other non-medical treatments, such as angiotensin receptor-neprilysin inhibitors and sodium-glucose cotransporter-2 inhibitors, for the medical management of AHF.

Another consideration is the impact of applying severity weights as recommended by NICE on the study results, although the severity-weighted estimates did not change the cost-effectiveness findings of this evaluation. NICE defined the weight groups based on how the end-of-life criteria were applied in previous appraisals, and there is no evidence on whether this truly reflects societal preferences [16]. In addition, the QALY shortfall cut-offs are higher than those used in other countries, such as the Netherlands (0.70) [17]. Further research is needed to understand the appropriateness and potential implications of the severity-weightings on cost-effectiveness decisions for different health technologies and interventions.

The study’s conclusions are based on the UK healthcare system and may not be applicable to other healthcare systems. For example, in settings where ongoing costs for medical management is substantially higher LVADs might be considered cost-effective compared to medical management. Similarly, the analyses are based on currently available LVADs, and they may not be applicable to future LVAD technologies. Should the risk of major events and complications reduce with next generation devices, this is likely to impact on the results of subsequent cost-effectiveness analyses.

This study has important implications for research and policy both in the UK and internationally. It presents a robust framework for analysing the cost-effectiveness of therapies for patients with AHF. The study also highlights the need for more evidence around medical management and the impacts of INTERMACS profiles on cost-effectiveness estimates in this patient population.

## Supporting information

S1 File Model inputs. (DOCX)

## References

[1] Clegg A., et al., The clinical and cost-effectiveness of left ventricular assist devices for end-stage heart failure: a systematic review and economic evaluation. Health technology assessment 2005. 9(1): p. 1–132. doi: 10.3310/hta9450 16303098

[2] National Institute for Health and Care Excellence [NICE], Chronic heart failure in adults: diagnosis and management. 2018. 30645061

[3] Stewart S., et al., The current cost of heart failure to the National Health Service in the UK. European Journal of Heart Failure, 2002. 4(3): p. 361–371. doi: 10.1016/s1388-9842(01)00198-2 12034163

[4] Urbich M., et al., A systematic review of medical costs associated with heart failure in the USA (2014–2020). Pharmacoeconomics, 2020. 38: p. 1219–1236. doi: 10.1007/s40273-020-00952-0 32812149 PMC7546989

[5] National Institute for Health and Care Excellence [NICE], Short-term circulatory support with left ventricular assist devices as a bridge to cardiac transplantation or recovery, in Interventional procedures guidance [IPG177]. 2006, National Institute for Health and Care Excellence: United Kingdom.

[6] Colombo P.C., et al., Comprehensive Analysis of Stroke in the Long-Term Cohort of the MOMENTUM 3 Study. Circulation, 2019. 139(2): p. 155–168. doi: 10.1161/CIRCULATIONAHA.118.037231 30586698

[7] Beese S., Saygin Avsar T., Price M., Quinn D., Lim H. S., Dretzke J., et al. (Accepted/In press)., Clinical and Cost-effectiveness of Left Ventricular Assist Devices as Destination Therapy for Advanced Heart Failure: Systematic Review and Economic Evaluation. 2024: Health Technology Assessment. 10.3310/MLFA4009 PMC11367304 39189844

[8] National Institute for Health and Care Excellence [NICE]. NICE health technology evaluations: the manual. 2022 17.05.2022]; Available from: https://www.nice.org.uk/process/pmg9/resources/guide-to-the-methods-of-technology-appraisal-2013-pdf-2007975843781.

[9] Schueler S., et al., Cost‐effectiveness of left ventricular assist devices as destination therapy in the United Kingdom. ESC heart failure, 2021. 8(4): p. 3049–3057. doi: 10.1002/ehf2.13401 34047072 PMC8318455

[10] Lim H.S., et al., A Clinical and Cost-effectiveness Analysis of The HeartMate 3 Left Ventricular Assist Device for Transplant-ineligible Patients: A United Kingdom Perspective. The Journal of Heart and Lung Transplantation, 2022. doi: 10.1016/j.healun.2021.11.014 34922821

[11] ONS, National life tables–life expectancy in the UK: 2018 to 2020. 2021.

[12] Flack S., Taylor M., and Trueman P., Cost-Effectiveness of Interventions for Smoking Cessation. 2007, York Health Economics Consortium

[13] Rose E.A., et al., Long-Term Use of a Left Ventricular Assist Device for End-Stage Heart Failure. New England Journal of Medicine, 2001. 345(20): p. 1435–1443. doi: 10.1056/NEJMoa012175 11794191

[14] Goldstein D.J., et al., Association of Clinical Outcomes With Left Ventricular Assist Device Use by Bridge to Transplant or Destination Therapy Intent: The Multicenter Study of MagLev Technology in Patients Undergoing Mechanical Circulatory Support Therapy With HeartMate 3 (MOMENTUM 3) Randomized Clinical Trial. JAMA Cardiology, 2020. 5(4): p. 411–419. doi: 10.1001/jamacardio.2019.5323 31939996 PMC6990746

[15] McNamara S., et al., Quality-Adjusted Life Expectancy Norms for the English Population. Value in Health, 2022. doi: 10.1016/j.jval.2022.07.005 35965226

[16] Angelis A., et al., The evolving nature of Health Technology Assessment: a critical appraisal of NICE’s new methods manual. Value in Health, 2023. doi: 10.1016/j.jval.2023.05.015 37268059

[17] Skedgel C., et al., Considering Severity in Health Technology Assessment: Can We Do Better? Value in Health, 2022. 25(8): p. 1399–1403. doi: 10.1016/j.jval.2022.02.004 35393254

