# Correction: Cost-effectiveness of Left Ventricular Assist Devices (LVADs) as destination therapy in the UK: An economic modelling study

**DOI:** 10.1371/journal.pone.0321324

**Published:** 2025-04-01

**Authors:** Tuba Saygın Avşar, Louise Jackson, Pelham Barton, Sophie Beese, Hoong S. Lim, David Quinn, Malcolm Price, David J. Moore

The eighth author’s name is spelled incorrectly. The correct name is: David J. Moore.

An affiliation is missing for the seventh author. Malcolm Price is also affiliated with #2: University, of Birmingham, Birmingham, United Kingdom.
