# Supplementary material for: Cost-effectiveness of Left Ventricular Assist Devices (LVADs) as destination therapy in the UK: An economic modelling study
Source: PLoS One. 2024 Dec 4;19(12):e0312912. doi: 10.1371/journal.pone.0312912 (PMC11616806; doi:10.1371/journal.pone.0312912)
Supplement: S1 File — (DOCX) [file pone.0312912.s001.docx]

# Cost-effectiveness of Left Ventricular Assist Devices (LVADs) as destination therapy in the UK: An economic modelling study

## Model inputs

Saygın Avşar T^1^, Jackson L^2^, Barton P^3^, Beese S^4^, Lim HS^5^, Quinn D^6^, Price M^7^, Moore DJ^8^

^1^PhD, Department of Applied Health Research, University College London, London, United Kingdom, ORCID ID: 0000-0002-4143-3852

^2^PhD, Jackson L, University of Birmingham, Birmingham, United Kingdom, ORCID ID: 0000-0001-8492-0020

^3^ PhD, University of Birmingham, Birmingham, United Kingdom, ORCID ID: 0000-0001-8936-3745

^4^ PhD, University of Birmingham, Birmingham, United Kingdom, ORCID ID: 0000-0001-6329-0779

^5^ PhD, University of Birmingham Hospitals, Birmingham, United Kingdom, ORCID ID: 0000-0002-6569-1805

^6^ PhD, University of Birmingham Hospitals, Birmingham, United Kingdom, ORCID ID: ORCID ID: 0000-0003-2465-305X

^7^ PhD, University of Birmingham, Birmingham, United Kingdom, ORCID ID: 0000-0002-7352-3027

^8^ PhD, University of Birmingham, Birmingham, United Kingdom, ORCID ID: 0000-0002-4163-4080

**Correspondence:**

Tuba Saygın Avşar, [t.avsar@ucl.ac.uk](mailto:t.avsar@ucl.ac.uk) , twitter @saygnavsartuba

Supplementary Table 1. Probabilities and health utilities used in the model

| Parameters | Mean/month | SE | Source (Trial/  Database) |
| --- | --- | --- | --- |
| Mortality | | | |
| Mortality risk in LVAD (Mehra et al., 2021)  1-6 months  7-12 months  13-24 months  25+ months | 0.021  0.008  0.007  0.007 | 0.0009  0.0002  0.0004  0.0004 | MOMENTUM |
| Mortality risk in MM (Rose et al., 2001) | 0.085 | 0.0085 | REMATCH |
| Morbidity | | | |
| First stroke in LVAD patients (Kirklin et al., 2020)  1-3 months  4+ months | 0.017  0.003 | 0.0059  0.0022 | INTERMACS |
| Probability of a second stroke after a non-disabling stroke (Kirklin et al., 2020) | 0.002 | 0.0016 | Same as above |
| Proportion of disabling stroke in patients experiencing stroke (Milano et al., 2018) | 0.28 | 0.028 | ENDURANCE DT |
| Right Heart Failure (RHF) in LVAD patients  1 month (Teuteberg et al., 2020) – early RHF  2+ months (Teuteberg et al., 2020) – hospitalisation due to late RHF | 0.140  0.002 | 0.0067  0.0010 | INTERMACS 9^th^ |
| Proportion requiring RVAD placement in patients with LVAD (Rogers et al., 2017) | 0.125 | 0.0523 | ENDURANCE DT |
| Severe aortic regurgitation (AR) in LVAD patients (Jorde et al., 2014) | 0.004 | 0.0048 | Columbia Uni Med. Centre |
| Proportion of AR patients requiring operation for valve replacement (Jorde et al., 2014) | 0.33 | 0.0355 | Same as above |
| Stroke in MM patients (Homma et al., 2012) | 0.001 | 0.0007 | WARCEF trial |
| Gastrointestinal Bleeding (GIB) in LVAD patients – *12m follow-up*  1-3 months (includes surgical bleeding)  4+ months | 0.032  0.010 | 0.0043  0.0003 | (Meta-analysis) |
| Driveline infection (DI) in LVAD patients | 0.011 | 0.0001 | (Meta-analysis) |
| Pump infection (PI) in LVAD patients (Tattevin et al., 2019)  1^st^ month  2+ months | 0.008  0.003 | 0.0035  0.0021 | ASSIST-ICD 19 centres |
| Pump exchange in LVAD patients (any reason) (Kirklin et al., 2017) - *device malfunctions over 72 m* | 0.002 | 0.0003 | INTERMACS |
| Arrhythmia in LVAD patients – *12m follow-up* | 0.018 | 0.0015 | (Meta-analysis) |
| Probability of re-admission apart from stroke in OMM patients (Ambardekar et al., 2019) | 0.068 | 0.0356 | MEDAMACS |
| Mortality within 30 days of major events |  |  |  |
| Death due to stroke in LVAD patients (Milano et al., 2018) – *12m follow-up* | 0.25 | 0.0475 | ENDURANCE |
| Death due to stroke in MM patients** (Freeman et al., 2011) | 0.085 | 0.013 | NA |
| Death due to early RHF^++^ | 0.021 | 0.0012 | Expert view |
| Death due to AR^++^ | 0.021 | 0.0012 | Expert view |
| Long-term mortality in patients with major events (monthly) | | | |
| Mortality risk in disabling stroke survivors (Kirklin et al., 2020) | 0.024 | 0.0119 | INTERMACS |
| Mortality risk in AR survivors (Truby et al., 2018) | 0.021 | 0.0112 | INTERMACS |
| Mortality risk in RHF survivors | 0.085 | 0.013 | Expert view |
| Quality of life (Health utility) | | | |
| Utility in DT (LVAD)  1m  2m-6m  7m-12m  13m+ | 0.51  0.76  0.77  0.77 | 0.014 0.011  0.010  0.014 | MOMENTUM |
| Utility in MM | 0.51 | 0.014 | MOMENTUM |
| Utility loss after stroke (Chaisinanunkul et al., 2015; Post Piet et al., 2001) | 0.11 | 0.0255 | NA |
| Utility loss after disabling stroke (Chaisinanunkul et al., 2015; Post Piet et al., 2001) | 0.67 | 0.067 | Same as above |
| Utility in patients experiencing RHF^+^ | 0.405 | 0.0120 | Expert view |
| Utility in patients experiencing AR^++^ | 0.405 | 0.0120 | Expert view |
| Utility loss after GIB (Silvestry et al., 2019) | 0.048 | 0.0048 | Expert view & previous model |
| Utility loss after DI and PI (Long et al., 2014) | 0.156 | 0.0156 | Expert view & previous model |
| Utility loss after PE (Silvestry et al., 2019) | 0.24 | 0.024 | Expert view & previous model |
| Utility loss after arrythmia  Utility loss after AF (Witassek et al., 2019)  Utility loss after VF (Mark et al., 2008)  (0.58 of all arrythmia cases were assumed to be VF) (Mehra et al., 2019) | 0.012  0.063 | 0.001  0.006 | MOMENTUM |
| Note: In the probabilistic sensitivity analysis (PSA), beta distribution was used for all the probabilities and health utilities. Random sampling method was used to generate the parameters in the PSA unless the parameter was time-dependent in which case the difference method was used. | | | |

Supplementary Table 2. Cost inputs used in the model

| One-off cost items | Currency codes | Cost  (2019) | SE |
| --- | --- | --- | --- |
| Complex LVAD implant cost  (applied for 10% of the LVAD patients) | ED08Z | £130,914 | £13,091 |
| Standard LVAD implant  (applied for 90% of the LVAD patients) | ED09Z | £90,484 | £9,048 |
| Average LVAD implant cost | ED08Z/9Z | £94,527 | £9,453 |
| Complex heart transplantation | ED04Z | £61,070 | £6,011 |
| Stroke | AA35A-F | £3,417 | £341 |
| Right heart failure | EB03A-E | £1,972 | £197 |
| Aortic valve replacement for aortic regurgitation | ED24A-ED25C | £12,928 | £1,292 |
| Gastrointestinal bleeding | FD03A-H | £1,235 | £124 |
| Driveline infection and pump infection | HE81A-C | £3,478 | £348 |
| Pump exchange for any reason (assumed to be same as average LVAD implantation) | ED08Z | £94,527 | £9,453 |
| RVAD placement (operation cost assumed to be same as DI) | NA | £13,740 | £1,374 |
| Arrhythmia | EB07A-E | £952 | £95 |
| Death [36] | NA | £9,775 | £255 |
| Monthly ongoing costs | |  |  |
| Monthly cost for LVAD patients (outpatient) (Chew et al. 2017) | NA | £958 | £244 |
| Monthly outpatient costs for OMM patients (Clegg et al.2007) | NA | £644 | £64 |
| Cost per re-admission apart from stroke in OMM patients (Clegg et al.2007, Girling et al.2007) | NA | £3,389 | £339 |

Supplementary Table 3. Values used in one-way sensitivity analysis

| Parameter | Value1 | Source | Base-case | Value2 | Source |
| --- | --- | --- | --- | --- | --- |
| LVAD implantation cost | £91,162 | (Schueler et al., 2020) | £94,527 | £109,140 | (Lim et al., 2022) |
| End-of-life care cost is doubled for MM | - | - | £9,775 | £19,550 | Assumption |
| Monthly outpatient costs in MM patients (outpatient) | £72 | (Lim et al., 2022) | £644 | £2,951 | (Silvestry et al., 2019) |
| Outpatient costs for LVAD patients | £72 | (Lim et al., 2022) | £958 | £1,952 | (Clegg et al., 2007) |
| Cost per readmission per MM patient | £2,711 | 0.80*£3,389 | £3,389 | £9,041 | (Baras Shreibati et al., 2017) |
| Proportion of RHF patients receiving RVAD after an LVAD | 0.116 | (Kirklin et al., 2017) | 0.125 | 0.138 | Assumption |
| Probability of RHF hospitalisation after the second month | 0.001 | 0.002/2 | 0.002 | 0.004 | 0.002*2 |
| Probability of severe AR | 0.002 | 0.004/2 | 0.004 | 0.008 | 0.004*2 |
| Probability of stroke in LVAD patients | 0.008 & 0.003 | (Starling et al., 2017) | 0.017 & 0.003 | 0.017 & 0.008 | (Starling et al., 2017) |
| Probability of stroke in MM patients | - | - | 0.001 | 0.002 | (Baras Shreibati et al., 2017) |
| GIB in LVAD recipients | 0.016 & 0.40 | (Kirklin et al., 2017) | 0.032 & 0.010 | 0.04 & 0.06 | (Kirklin et al., 2017) |
| Driveline infection in LVAD recipients | 0.006 | 0.011/2 | 0.011 | 0.024 | (Tattevin et al., 2019) |
| Utility loss after disabling stroke | 0.450 | (Schueler et al., 2020) | 0.670 | 0.7 | 0.67*1.05 |
| Utility loss after non-disabling stroke | 0.09 | (0.11/0.14)*0.11 | 0.11 | 0.14 | (Luengo-Fernandez et al., 2013) |
| Utility in MM patients | 0.40 | (Baras Shreibati et al., 2017) | 0.51 | 0.64 | (Silvestry et al., 2019) |
| Utility in LVAD recipients (12m) | 0.70 | (Baras Shreibati et al., 2017) | 0.77 | 0.85 | (Chew et al., 2017) |
| MM mortality risk | 0.070 | (Schueler et al., 2020) | 0.085 | 0.09 | 0.085*1.05 |
| Reduced LVAD mortality risk after 12m | 0.005 | (Lim et al., 2022) | 0.021 & 0.012 | 0.011 | (Mehra et al., 2021) |

References

Ambardekar, A. V., Kittleson, M. M., Palardy, M., Mountis, M. M., Forde-McLean, R. C., DeVore, A. D., . . . Stewart, G. C. (2019). Outcomes with ambulatory advanced heart failure from the Medical Arm of Mechanically Assisted Circulatory Support (MedaMACS) Registry. *The Journal of Heart and Lung Transplantation*, *38*(4), 408-417. <https://doi.org/https://doi.org/10.1016/j.healun.2018.09.021>

Baras Shreibati, J., Goldhaber-Fiebert, J. D., Banerjee, D., Owens, D. K., & Hlatky, M. A. (2017). Cost-Effectiveness of Left Ventricular Assist Devices in Ambulatory Patients With Advanced Heart Failure. *JACC Heart Fail*, *5*(2), 110-119. <https://doi.org/10.1016/j.jchf.2016.09.008>

Chaisinanunkul, N., Adeoye, O., Lewis, R. J., Grotta, J. C., Broderick, J., Jovin, T. G., . . . Additional contributors from, D. T. S. C. (2015). Adopting a Patient-Centered Approach to Primary Outcome Analysis of Acute Stroke Trials Using a Utility-Weighted Modified Rankin Scale. *Stroke*, *46*(8), 2238-2243. <https://doi.org/10.1161/STROKEAHA.114.008547>

Chew, D. S., Manns, B., Miller, R. J. H., Sharma, N., & Exner, D. V. (2017). Economic Evaluation of Left Ventricular Assist Devices for Patients With End Stage Heart Failure Who Are Ineligible for Cardiac Transplantation. *Canadian Journal of Cardiology*, *33*(10), 1283-1291. <https://doi.org/10.1016/j.cjca.2017.07.012>

Clegg, A. J., Scott, D. A., Loveman, E., Colquitt, J. L., Royle, P., & Bryant, J. (2007). Clinical and cost-effectiveness of left ventricular assist devices as a bridge to heart transplantation for people with end-stage heart failure: a systematic review and economic evaluation. *Eur Heart J*, *27*(24), 2929-2938. <https://doi.org/10.1093/eurheartj/ehi857>

Freeman, J., Zhu, R. P., Owen, D. K., & A.M., G. (2011). Cost-Effectiveness of Dabigatran Compared With Warfarin for Stroke Prevention in Atrial Fibrillation. *Ann Intern Med*, *154*(1), 1-11. <https://doi.org/10.7326/0003-4819-154-1-201101040-00289> %m 21041570

Homma, S., Thompson, J. L. P., Pullicino, P. M., Levin, B., Freudenberger, R. S., Teerlink, J. R., . . . Buchsbaum, R. (2012). Warfarin and Aspirin in Patients with Heart Failure and Sinus Rhythm. *New England Journal of Medicine*, *366*(20), 1859-1869. <https://doi.org/10.1056/NEJMoa1202299>

Jorde, U. P., Uriel, N., Nahumi, N., Bejar, D., Gonzalez-Costello, J., Thomas, S. S., . . . Naka, Y. (2014). Prevalence, Significance, and Management of Aortic Insufficiency in Continuous Flow Left Ventricular Assist Device Recipients. *Circulation: Heart Failure*, *7*(2), 310-319. <https://doi.org/doi:10.1161/CIRCHEARTFAILURE.113.000878>

Kirklin, J. K., Naftel, D. C., Myers, S. L., Pagani, F. D., & Colombo, P. C. (2020). Quantifying the impact from stroke during support with continuous flow ventricular assist devices: An STS INTERMACS analysis. *The Journal of Heart and Lung Transplantation*, *39*(8), 782-794. <https://doi.org/https://doi.org/10.1016/j.healun.2020.04.006>

Kirklin, J. K., Pagani, F. D., Kormos, R. L., Stevenson, L. W., Blume, E. D., Myers, S. L., . . . Naftel, D. C. (2017). Eighth annual INTERMACS report: Special focus on framing the impact of adverse events. *J Heart Lung Transplant*, *36*(10), 1080-1086. <https://doi.org/10.1016/j.healun.2017.07.005>

Lim, H. S., Shaw, S., Carter, A. W., Jayawardana, S., Mossialos, E., & Mehra, M. R. (2022). A Clinical and Cost-effectiveness Analysis of The HeartMate 3 Left Ventricular Assist Device for Transplant-ineligible Patients: A United Kingdom Perspective. *The Journal of Heart and Lung Transplantation*. <https://doi.org/10.1016/j.healun.2021.11.014>

Long, E. F., Swain, G. W., & Mangi, A. A. (2014). Comparative survival and cost-effectiveness of advanced therapies for end-stage heart failure. *Circ Heart Fail*, *7*(3), 470-478. <https://doi.org/10.1161/CIRCHEARTFAILURE.113.000807>

Luengo-Fernandez, R., Gray, A. M., Bull, L., Welch, S., Cuthbertson, F., Rothwell, P. M., & Oxford Vascular, S. (2013). Quality of life after TIA and stroke: ten-year results of the Oxford Vascular Study. *Neurology*, *81*(18), 1588-1595. <https://doi.org/10.1212/WNL.0b013e3182a9f45f>

Mark, D. B., Anstrom, K. J., Sun, J. L., Clapp-Channing, N. E., Tsiatis, A. A., Davidson-Ray, L., . . . Sudden Cardiac Death in Heart Failure Trial, I. (2008). Quality of life with defibrillator therapy or amiodarone in heart failure. *The New England journal of medicine*, *359*(10), 999-1008. <https://doi.org/10.1056/NEJMoa0706719>

Mehra, M. R., Cleveland Jr, J. C., Uriel, N., Cowger, J. A., Hall, S., Horstmanshof, D., . . .  on behalf of the, M. I. (2021). Primary results of long-term outcomes in the MOMENTUM 3 pivotal trial and continued access protocol study phase: a study of 2200 HeartMate 3 left ventricular assist device implants [<https://doi.org/10.1002/ejhf.2211>]. *European Journal of Heart Failure*, *23*(8), 1392-1400. <https://doi.org/https://doi.org/10.1002/ejhf.2211>

Mehra, M. R., Uriel, N., Naka, Y., Cleveland, J. C., Yuzefpolskaya, M., Salerno, C. T., . . . Goldstein, D. J. (2019). A Fully Magnetically Levitated Left Ventricular Assist Device — Final Report. *New England Journal of Medicine*, *380*(17), 1618-1627. <https://doi.org/10.1056/NEJMoa1900486>

Milano, C. A., Rogers, J. G., Tatooles, A. J., Bhat, G., Slaughter, M. S., Birks, E. J., . . . Pagani, F. D. (2018). HVAD: The ENDURANCE Supplemental Trial. *JACC: Heart Failure*, *6*(9), 792. <https://doi.org/10.1016/j.jchf.2018.05.012>

Post Piet, N., Stiggelbout Anne, M., & Wakker Peter, P. (2001). The Utility of Health States After Stroke. *Stroke*, *32*(6), 1425-1429. <https://doi.org/10.1161/01.STR.32.6.1425>

Rogers, J. G., Pagani, F. D., Tatooles, A. J., Bhat, G., Slaughter, M. S., Birks, E. J., . . . Milano, C. A. (2017). Intrapericardial Left Ventricular Assist Device for Advanced Heart Failure. *New England Journal of Medicine*, *376*(5), 451-460. <https://doi.org/10.1056/NEJMoa1602954>

Rose, E. A., Gelijns, A. C., Moskowitz, A. J., Heitjan, D. F., Stevenson, L. W., Dembitsky, W., . . . Meier, P. (2001). Long-Term Use of a Left Ventricular Assist Device for End-Stage Heart Failure. *New England Journal of Medicine*, *345*(20), 1435-1443. <https://doi.org/10.1056/NEJMoa012175>

Schueler, S., Silvestry, S., Cotts, W., Levy, W. C., Cheng, L. K., Villinger, J., . . . Mahr, C. (2020). Cost-Effectiveness of a Small Intrapericardial Centrifugal LVAD versus Medical Management in Destination Therapy Patients in the UK. *The Journal of Heart and Lung Transplantation*, *39*(4).

Silvestry, S., Mahr, C., Slaughter, M., Levy, W., Cheng, R., May, D., . . . Cotts, W. (2019). Cost-Effectiveness of a Small Intrapericardial Centrifugal LVAD versus Medical Management and Heart Transplantation. *Journal of Heart and Lung Transplantation*, *38*.

Starling, R. C., Estep, J. D., Horstmanshof, D. A., Milano, C. A., Stehlik, J., Shah, K. B., . . . Rogers, J. G. (2017). Risk Assessment and Comparative Effectiveness of Left Ventricular Assist Device and Medical Management in Ambulatory Heart Failure Patients: The ROADMAP Study 2-Year Results. *JACC: Heart Failure*, *5*(7), 518-527. <https://doi.org/https://doi.org/10.1016/j.jchf.2017.02.016>

Tattevin, P., Flécher, E., Auffret, V., Leclercq, C., Boulé, S., Vincentelli, A., . . . Galand, V. (2019). Risk factors and prognostic impact of left ventricular assist device–associated infections. *American Heart Journal*, *214*, 69-76. <https://doi.org/https://doi.org/10.1016/j.ahj.2019.04.021>

Teuteberg, J. J., Cleveland, J. C., Jr., Cowger, J., Higgins, R. S., Goldstein, D. J., Keebler, M., . . . Atluri, P. (2020). The Society of Thoracic Surgeons Intermacs 2019 Annual Report: The Changing Landscape of Devices and Indications. *The Annals of Thoracic Surgery*, *109*(3), 649-660. <https://doi.org/10.1016/j.athoracsur.2019.12.005>

Truby, L. K., Garan, A. R., Givens, R. C., Wayda, B., Takeda, K., Yuzefpolskaya, M., . . . Topkara, V. K. (2018). Aortic Insufficiency During Contemporary Left Ventricular Assist Device Support: Analysis of the INTERMACS Registry. *JACC. Heart failure*, *6*(11), 951-960. <https://doi.org/10.1016/j.jchf.2018.07.012>

Witassek, F., Springer, A., Adam, L., Aeschbacher, S., Beer, J. H., Blum, S., . . . Swiss, A. F. s. i. (2019). Health-related quality of life in patients with atrial fibrillation: The role of symptoms, comorbidities, and the type of atrial fibrillation. *PloS one*, *14*(12), e0226730-e0226730. <https://doi.org/10.1371/journal.pone.0226730>
